# Replacing the nutrients in dairy foods with non-dairy foods will increase cost, energy intake and require large amounts of food: National Health and Nutrition Examination Survey 2011–2014

**DOI:** 10.1017/S1368980020001937

**Published:** 2022-02

**Authors:** Christopher J Cifelli, Nancy Auestad, Victor L Fulgoni

**Affiliations:** 1 National Dairy Council, Rosemont, IL 60018-5616, USA; 2 Nutrition Insights LLC, St George, UT 84770, USA; 3 Nutrition Impact, LLC, Battle Creek, MI 49014, USA

**Keywords:** National Health and Nutrition Examination Survey, Dairy, Shortfall nutrients, Nutrient adequacy, Linear programming optimisation

## Abstract

**Objective:**

The US Dietary Guidelines for Americans recommends increased consumption of the dairy group to three daily servings for ages 9+ years to help achieve adequate intakes of prominent shortfall nutrients. Identifying affordable, consumer-acceptable foods to replace dairy’s shortfall nutrients is important especially for people who avoid dairy.

**Design:**

Linear programming identified food combinations to replace dairy’s protein and shortfall nutrients. We examined cost, energy and dietary implications of replacing dairy with food combinations optimised for lowest cost, fewest kJ or the smallest amount of food by weight.

**Setting:**

National Health and Nutrition Examination Survey (2011–2014).

**Participants:**

Nationally representative sample of US population; 2 years and older (*n* 15 830).

**Results:**

Phase 1 (only dairy foods excluded): when optimised for lowest cost or fewest kJ, all non-dairy food replacements required large amounts (2·5–10 cups) of bottled/tap water. Phase 2 (dairy and unreasonable non-dairy foods excluded (e.g. baby foods; tap/bottled water): when intake of non-dairy foods was constrained to <90th percentile of current intake, the lowest cost food combination replacements for dairy cost 0·5 times more and provide 5·7 times more energy; the lowest energy food combinations cost 5·9 times more, provide 2·5 times more energy and require twice the amount of food by weight; and food combinations providing the smallest amount of food by weight cost 3·5 times more and provide five times more energy than dairy.

**Conclusions:**

Identifying affordable, consumer-acceptable foods that can replace dairy’s shortfall nutrients at both current and recommended dairy intakes remains a challenge.

Dietary patterns are shaped by the amounts and types of foods consumed^(1)^. The US Dietary Guidelines for Americans (DGA) encourages healthy eating to support nutrient adequacy, a healthy body weight and reduced risk of chronic disease^(2)^. Many Americans, however, continue to consume too much energy and insufficient amounts of dietary essential nutrients. This occurs, in part, because taste preferences, food costs, convenience, cultural influences, nutrition and health goals and increasingly environmental considerations influence individual food choices^(1,3)^.

Across the globe, dietary patterns for many do not meet nutrient recommendations^(4)^. In the USA, the 2015–2020 DGA highlighted ten *shortfall* nutrients, those with current intakes that are below recommended intakes: Ca, choline, dietary fibre, Fe, Mg, K and vitamins A, D, E and C^(2)^. Among these, Ca, vitamin D, K and dietary fibre were called *nutrients of public health concern* due to adverse health outcomes associated with their underconsumption. The dairy group is recognised as a significant dietary source of protein and many micronutrients, including six of the shortfall nutrients (Ca, vitamin D, K, vitamin A, Mg and choline) as well as P, riboflavin, vitamin B_12_, Zn and Se. At current intakes, dairy foods provide half of the Ca, nearly 60 % of vitamin D and about 15–25 % of the daily intake of K, vitamin A, vitamin B_12_, P, Zn, riboflavin and protein in the diet of Americans^(5)^. Increasing consumption of dairy foods to meet daily recommended amounts could lead to a lower prevalence of inadequate intakes of Ca, vitamin D, Mg, vitamin A and to a higher K intake^(5–7)^.

Concerns about environmental impacts of animal-based foods and the ability of agriculture to support an estimated 9–10 billion people on the planet by 2050 have led some to recommend reducing or eliminating meat or both meat and dairy from the diet^(8–10)^. The widely accepted FAO definition of sustainable diets states that they must support human health, be nutritionally adequate, culturally acceptable and affordable while optimising use of natural and human resources and protecting biodiversity and ecosystems^(11)^. While much of the research on sustainable diets has focused solely on the environmental impacts of diets, more research is needed that simultaneously examines the health, environmental, social and economic aspects.

Some people choose to avoid dairy foods because of allergies, cultural practices or for personal reasons^(12,13)^. Consequently, dietary deficiencies of one or more shortfall nutrients likely will occur unless they can be obtained from non-dairy foods. Diet modelling studies by US 2015 Dietary Guidelines Advisory Committee show that removing dairy from an otherwise nutritionally adequate diet would lower intakes of Ca by 68–88 %, vitamin D by 20–30 %, K by 15 %, vitamin A by 29–33 % in older adults and Mg by 31 % in older men^(14)^. The feasibility of non-dairy foods to provide Ca in an amount equivalent to that in a United States Department of Agriculture (USDA) cup-equivalent of dairy (53 % fat-free milk, 45 % low-fat cheese and 2 % yogurt) was subsequently examined^(14)^. At Ca intakes corresponding to that in the recommended 3 servings of dairy for ages ≥9 years, individual foods, such as Ca-fortified orange juice, leafy greens or canned salmon with bones, would provide too much energy and/or require consumption of excessively large amounts of food^(12,14)^.

Diet optimisation with linear programming is increasingly being used to optimise diets on two or more dimensions (e.g. cost, nutrition, health, cultural acceptability, environmental impacts)^(15,16)^. The broad range of scenarios that have been examined provides insights into designing dietary patterns that are composed of familiar foods, meet dietary guidance and nutrient requirements, are affordable across socio-economic strata, and/or address a multitude of environmental and other sustainability criteria. While some studies show that sustainable dietary patterns are possible with modest changes in eating patterns^(17–20)^, others point to trade-offs that would need to be addressed^(21–26)^, especially when dairy and/or meat are excluded from the diet^(24,26)^.

The aim of the current study was to identify non-dairy food combinations that could replace the protein and shortfall nutrients provided by dairy without increasing the cost, energy intake or the amount (g) of food needed to be consumed when dairy foods are excluded.

## Materials and methods

We used linear programming methodology to identify combinations of non-dairy foods that are consumed in the USA to replace the protein and shortfall nutrients otherwise provided in diets without dairy while minimising (i) cost, (ii) energy (kJ) and/or (iii) the amount of food by weight. We based our analyses on the energy content and nutrients in one USDA cup-equivalent of dairy. The cost, energy and consumption implications were examined at the US current average of 1·8 dairy servings for ages ≥2 years and the DGA recommended 3 daily servings for those 9 years and older. We also examined differences in the levels of protein, shortfall nutrients and limiting nutrients between USDA servings of dairy and the optimised food combinations (see online supplementary material, Supplemental Tables 1 and 2) but did not evaluate the implications of these differences to the overall nutritional quality of the diet.

### Dietary data source

The food and nutrient data source for each of the linear programming scenarios was the dietary component (*What We Eat In America* (WWEIA)) of the National Health and Nutrition Examination Survey (NHANES), which includes approximately 8000 foods (150 food categories) that are consumed in the USA. The dietary intake data were obtained from the first of the two 24-h recalls in the 2011–2012 and 2013–2014 NHANES data sets for persons 2 years and older (*n* 15 830), excluding data for pregnant and lactating women (*n* 184) and incomplete data (*n* 2643). The 24-h recall data for individual participants in these surveys include a description of individual foods and beverages consumed on the previous day (midnight to midnight) and the amount by weight. Complete descriptions of the dietary interview methods for NHANES are provided elsewhere^(27)^.

The USDA dairy composite used in the current study is the same as that used by the 2015 Dietary Guideline Advisory Committee in the diet modelling research described above (i.e. 53 % fat-free milk, 45 % low-fat cheese and 2 % yogurt)^(14)^. Table 1 shows the nutrient composition of USDA cup-equivalent servings of dairy based on NHANES 2011–2014.

**Table 1:** Contribution of the dairy group in the United States Department of Agriculture (USDA) Healthy US-Style Eating Pattern* and the energy and nutrient content in one USDA cup-equivalent of the dairy reference for the optimisation study

Nutrients	Healthy US-Style Eating Pattern*	Optimisation study: dairy reference	
	Percentage intake from dairy group, 2000 kcal (8368 kJ)	Energy and nutrients in USDA cup-equivalents of dairy	
WWEIA source	NHANES 2009–2010	NHANES 2011–2014	
No. of USDA cup- equivalent of dairy group	Three cup-equivalents	One cup-equivalent	Three cup-equivalents
Energy	12 %	435 kJ	1305 kJ
Protein	29 %	9 g	27 g
Shortfall nutrients			
Nutrients of public health concern			
Ca	69 %	323 mg	968 mg
Vitamin D	65 %	2·8 µg	8·3 µg
K	21 %	382 mg	1146 mg
Dietary fibre	N/A	<0·1 g	0·1 g
Other shortfall nutrients			
Choline	21 %	40 mg	120 mg
Fe	2 %	0·1 mg	0·4 mg
Mg	17 %	28 mg	85 mg
Vitamin A	33 %	148 µg	445 µg
Vitamin C	N/A	0·3 mg	1·0 mg
Vitamin E	1 %	0·2 mg	0·7 mg
Nutrients to limit			
Saturated fat	9 %	1·0 g	3·0 g
Na	35 %	133 mg	399 mg
Added sugars	0 g	0 g	0 g

WWEIA, What We Eat In America; NHANES, National Health and Nutrition Examination Survey; N/A, not available.

* 2015 Dietary Guidelines Advisory Committee Report^(14)^.

### Cost data source

The details of the method to derive the cost data have been described previously^(28)^. Briefly, we used the food prices for all foods and beverages as reported in the 2001–2004 cycles of NHANES after adjusting for inflation since that time. The original cost database was developed by the USDA Center for Nutrition Promotion and Policy using the Nielsen Homescan Consumer Panel price data that were released in May 2008^(29,30)^. The monthly Consumer Price Index from the Bureau of Labor Statistics values^(30)^ was averaged over each 2-year NHANES cycle to adjust for inflation. When necessary, food codes for 2011–2014 USDA Food and Nutrient Database for Dietary Studies (FNDDS) data were matched to the most closely matching food codes in the 2001–2004 FNDDS. As described previously, mixed dishes prepared with multiple ingredients that did not correspond to single Bureau of Labor Statistics code were regressed using the Food Patterns Equivalents Database individual food components^(28)^. With the exception of bottled water, for which the cost value was not available, all food and beverage prices were derived as described. In our analyses, we used a cost of $US 0·25/l for bottled water (WWEIA category code, 7704).

### Linear programming analyses

Linear programming is a mathematical approach that is increasingly being used in dietary pattern research to optimise dietary components across a wide range of parameters and constraints^(15,16)^. We used linear programming to find unique combinations of non-dairy foods in the 2011–2014 WWEIA food categories that would substitute for the amount of protein and ten shortfall nutrients in one USDA cup-equivalent of dairy, the dairy component of the USDA Healthy US-Style Eating Pattern. The ten shortfall nutrients are Ca, choline, fibre, Fe, Mg, K, vitamin A, vitamin C, vitamin D and vitamin E. Table 1 shows the contribution of dairy to the intake of protein and the shortfall nutrients in a Healthy US-Style Eating Pattern^(14)^.

As described by Gazan *et al.* ^(16)^, the key parameters in mathematical optimisation are decision variables, objective function and constraints. Table 2 shows each of these as applied in the present optimisation study. After preliminary analyses, our study was conducted in two phases. In phase 1, only dairy foods, including the dairy component in mixed dishes, were excluded from the WWEIA food categories. In phase 2, based on our preliminary analyses and the results from phase 1, select foods and beverages that were not reasonable non-dairy options on a population basis also were excluded. These comprised the following categories (WWEIA category code): baby foods (9002, 9004, 9006, 9008, 9010, 9012, 9202), infant formulas (9402, 9404), protein and nutritional powders (9802), nutritional beverages (7208) and beverages with virtually no energy content, but with small amounts of micronutrients; that is, coffee (7302), tap water (7702), bottled water (7704), diet soft drinks (7102) and other diet drinks (7106). Additionally, within the three optimisation scenarios (i.e. for lowest cost, fewest kJ or smallest amount of food by weight), two levels of constraints for consumption amounts were examined: (i) no constraints and (ii) no more than the 90th percentile of current intake of non-dairy WWEIA foods.

**Table 2:** Linear regression optimisation modelling of non-dairy food replacements for select nutrients in one United States Department of Agriculture (USDA) cup-equivalent of dairy*

Optimisation parameter	Description
Decision variables	The amount of protein and the ten shortfall nutrients in one USDA cup-equivalent serving of dairy† from non-dairy foods and beverages
Population studied	Americans, ages 2+ years with exclusion for incomplete data and pregnant or lactating females (NHANES 2011–2014, day 1 dietary data)
Type of modelling	Population-based
Objective functions	Minimise the sum of the absolute relative deviations between the optimised and average observed quantities of foods/beverages consumed in the USA (2011–2014 NHANES) in accordance with constraints as described below
Data sources	Non-dairy foods and beverages and their energy and nutrient content: – WWEIA dietary component of NHANES 2011–2012 and 2013–2014 excluding all dairy products‡ Cost of non-dairy foods and beverages – USDA Food and Nutrition Service, Center for Nutrition Policy and Promotion Food Prices Database
Constraints for the WWEIA food categories (2011–2014) to replace select nutrients corresponding to those in one USDA cup-equivalent serving	Phase 1 Optimisation priorities: Scenario 1. Lowest cost non-dairy food combinations Scenario 2. Fewest kJ in non-dairy food combinations Scenario 3. Smallest amount of food by weight (g) non-dairy food combinations All three scenarios: Consumption constraints for WWEIA food categories: – None – No more than the 90th percentile of current consumption Phase 2 Same as scenario 1 with the following additional exclusions from the WWEIA food database (WWEIA Food Category): – Foods and beverages that are not reasonable replacement foods on a population basis: baby foods, protein and nutritional powders (9802); nutritional beverages (7208) – Beverages with virtually no energy (kJ), but that contain small amounts of micronutrients: coffee (7302), tap water (7702), bottled water (7704), diet soft drinks (7102) and other diet drinks (7106)
Software programme used	PROC OPTIMA

WWEIA, What We Eat In America; NHANES, National Health and Nutrition Examination Survey.

* Adapted from Gazan *et al*.^(16)^.

† One USDA cup-equivalent serving: 53 % fat-free milk, 45 % low-fat cheese and 2 % yogurt^(14)^.

‡ Dairy products excluded from the WWEIA database. WWEIA Food Category (category codes): low-fat milk/yogurt (1006, 1008, 1206, 1208, 1804, 1404), higher fat milk/yogurt (1002, 1004, 1202, 1204, 1402, 1802), cheese (1602, 1604) and cheese sandwiches (separate code) (3720).

SAS version 9.4 with survey parameters including primary sampling units, strata and dietary sample weights was used for these analyses. PROC OPTIMA was used for linear programming to obtain substitutions in three specific analyses that separately minimised food weight (g), energy (kJ) or cost and at the same time identified foods that contain the nutrients from 1 serving of USDA dairy foods within the two consumption constraints.

### Reference servings

The 2015–2020 DGA defines a serving size as ‘a standardized amount of a food, such as a cup or an ounce, used in providing information about a food within a food group, such as in dietary guidance. Serving size on the Nutrition Facts label is determined based on the Reference Amounts Customarily Consumed (RACC) for foods that have similar dietary usage, product characteristics, and customarily consumed amounts for consumers to make ‘like product’ comparisons’^(2)^. Table 3 shows the RACC serving sizes that we used to estimate the number of reference servings corresponding to the gram amounts of foods in the non-dairy food combinations. The US Food and Drug Administration (FDA) allows for flexibility within limits for the labelled servings on foods and beverages^(31)^; therefore, the number of RACC servings shown in Tables 4 and 5 represents an approximate number of servings.

**Table 3:** Reference data for converting non-dairy replacement foods and beverages to serving sizes

Optimisation study: foods/beverages	RACC*		
WWEIA food category	FDA reference category	One RACC serving	
		g or ml	Common measures
Eggs and omelettes	Egg and egg substitutes Egg mixtures, for example, egg foo young, scrambled eggs, omelettes	110 g	Approximately two eggs
Ready-to-eat cereal, higher sugar (>21·2 g/100 g)	Cereals and other grain products Breakfast cereals, ready-to-eat, weighing ≥20 g but <43 g/cup; high-fibre cereals containing ≥28 g of fibre/100 g	40 g	Approximately one cup
	Breakfast cereals, ready-to-eat, weighing ≥43 g/cup; biscuit types	60 g	Approximately ¾ cup
Rolls and buns	Breads (excluding sweet quick type), rolls	50 g	Approximately one small
Citrus juice	Fruits and fruit juice Juices, nectars, fruit drinks	240 ml	One cup (8 fl. oz)
Tap water; bottled water; coffee	Beverages Carbonated and noncarbonated beverages, wine coolers, water	360 ml	1 ½ cup (12 fl. oz)


Fish	Fish, shellfish, game meats and meat or poultry substitutes		
	Fish, shellfish or game meat, canned	85 g	About 3 oz
Nutrition bars	Bakery products		
	Grain-based bars with or without filling or coating, for example, breakfast bars, granola bars, rice cereal bars	40 g	Approximately one small nutrition bar
Carrots; dark green vegetables, excludes lettuce	Vegetables		
	Fresh or frozen	85 g	About half to one cup
	Vacuum packed	95 g	
	Canned in liquid, cream-style maize, canned or stewed tomatoes, pumpkin or winter squash:	130 g	
Lettuce and lettuce salads	Salads		
	All other salads, for example, egg, fish, shellfish, bean, fruit or vegetable salads	100 g	Approximately 1 ½ cups

WWEIA, What We Eat In America; RACC, Reference Amounts Customarily Consumed.

* *Source*: 21 FDA CFR 101.12 Reference Amounts Customarily Consumed per Eating Occasion^(31)^.

**Table 4:** Phase 1: Impact of optimising non-dairy food combinations to provide the protein and ten shortfall nutrients in United States Department of Agriculture (USDA) cup-equivalent servings of dairy at the lowest cost, fewest kJ or smallest amount of food by weight*

WWEIA food category	Non-dairy replacement foods	Cost	kJ	Weight (g)	No. of RACC servings	
	USDA servings of dairy	One cup-equivalent†			One cup- equivalent	1·8 cup- equivalents
Dairy reference		$US 0·41	435	250	1	1·8
Scenario 1. Optimised for lowest cost						
A. WWEIA consumption constraint: none						
2502	Eggs and omelettes	0·14	301	41	0·4	0·6
4602	Ready-to-eat cereal, higher sugar (>21·2 g/100 g)	0·30	745	48	0·8–1·2	1·4–2·0
7002	Citrus juice	0·10	209	106	0·4	0·7
7702	Tap water	0	0	5022	13·9	23·7
	Total	0·55	1259	5217		
	Ratio to dairy	1·34	2·88	20·86		
B. WWEIA consumption constraint: 90th percentile of non-dairy foods						
2502	Eggs and omelettes	0·03	54	8	0·1	0·1
4204	Rolls and buns	0·04	686	58	1·2	2·0
4602	Ready-to-eat cereal, higher sugar (>21·2 g/100 g)	0·43	1075	69	1·1–1·7	2·0–2·9
7002	Citrus juice	0·06	117	60	0·2	0·4
7702	Tap water	0	0	2577	7·2	12·2
	Total	0·56	1933	2772		
	Ratio to dairy	1·36	4·42	11·08		
Scenario 2. Optimised for fewest kJ						
A. WWEIA consumption constraint: none						
2402	Fish	0·85	356	49	0·6	1·0
6404	Carrots	0·05	29	17	0·2	0·3
7302	Coffee	0·10	25	344	1·0	1·6
7702	Tap water	0	0	9875	27·4	46·6
	Total	0·99	410	10 285		
	Ratio to dairy	2·41	0·94	41·12		
B. WWEIA consumption constraint: 90th percentile of non-dairy foods						
2402	Fish	0·85	356	49	0·6	0·9
6404	Carrots	0·05	29	17	0·2	0·3
7302	Coffee	0·10	25	344	1·0	1·6
7702	Tap water	0	0	2577	7·2	12·2
7704	Bottled water	0·63	0	2519	7·0	11·9
	Total	1·62	410	5506		
	Ratio to dairy	3·95	0·94	22·01		
Scenario 3. Optimised for smallest amount of foods by weight						
A. WWEIA consumption constraint: none						
4602	Ready-to-eat cereal, higher sugar (>21·2 g/100 g)	0·49	1205	77	1·3–1·9	2·2–3·3
5404	Nutrition bars	0·93	979	64	1·6	2·7
	Total	1·42	2184	141		
	Ratio to dairy	3·46	4·99	0·56		
B. WWEIA consumption constraint: 90th percentile of non-dairy foods						
4602	Ready-to-eat cereal, higher sugar (>21·2 g/100 g)	0·49	1205	77	1·3–1·9	2·2–3·3
5404	Nutrition bars	0·93	979	64	1·6	2·7
	Total	1·42	2184	141		
	Ratio to dairy	3·46	4·99	0·56		

RACC, Reference Amounts Customarily Consumed; WWEIA, What We Eat In America.

* See footnote of Table 1 for dairy products excluded from the WWEIA database.

† The values for cost, energy (kJ) and amount of food by weight correspond to those for one USDA cup-equivalent serving of dairy. To obtain the values corresponding to the current average of 1·8 dairy servings for ages ≥2 years and for the recommended 3·0 dairy servings for ages ≥9 years, multiply the values shown by 1·8 and 3·0, respectively. For example, under scenario 3 at intakes to replace the nutrients provided by 1·8 USDA cup-equivalents of dairy, the calculations for the daily cost of consuming *higher sugar ready-to-eat cereal* and *nutrition bars* and the number of kJ provided are $US 1·42 × 1·8 = $US 2·56 and 2184 kJ × 1·8 = 3931 kJ, respectively.

**Table 5:** Phase 2: Impact of optimising non-dairy food combinations to provide the protein and ten shortfall nutrients in United States Department of Agriculture (USDA) cup-equivalent servings of dairy at the lowest cost, fewest kJ or smallest amount of food by weight*

WWEIA food category	Non-dairy replacement foods	Cost	kJ	Weight (g)	No. of RACC Servings	
	USDA servings of dairy	One cup-equivalent			One cup-equivalent	1·8 cup-equivalents
Dairy reference		$US 0·41	435	250	1	1·8
Scenario 1. Optimised for lowest cost						
A. WWEIA consumption constraint: none						
2502	Eggs and omelettes	0·01	29	4	<0·1	0·1
4204	Rolls and buns	0·09	1536	131	2·6	4·5
4602	Ready-to-eat cereal, higher sugar (>21·2 g/100 g)	0·47	1172	75	1·3–1·9	2·1–3·2
7002	Citrus juice	<0·01	4	2	<0·1	<0·1
	Total	0·58	2736	212		
	Ratio to dairy	1·41	6·26	0·85		
B. WWEIA consumption constraint: 90th percentile of non-dairy foods						
2502	Eggs and omelettes	0·01	29	4	<0·1	0·1
4204	Rolls and buns	0·07	1238	105	2·1	3·6
4602	Ready-to-eat cereal, higher sugar (>21·2 g/100 g)	0·44	1092	70	1·2–1·7	2·0–3·0
7002	Citrus juice	0·07	142	71	0·3	0·5
	Total	0·60	2502	251		
	Ratio to dairy	1·45	5·72	1·00		
Scenario 2. Optimised for fewest kJ						
A. WWEIA consumption constraint: none						
2402	Fish	0·84	356	49	0·6	1·0
6408	Dark green vegetables, excludes lettuce	2·43	653	418	4·9	8·4
	Total	3·27	1008	467		
	Ratio to dairy	7·97	2·30	1·87		
B. WWEIA consumption constraint: 90th percentile of non-dairy foods						
2402	Fish	0·69	289	40	0·5	0·8
6408	Dark green vegetables, excludes lettuce	1·16	314	200	2·4	4·0
6410	Lettuce and lettuce salads	0·36	71	111	1·3	2·2
7002	Citrus juice	0·20	397	200	0·8	1·4
	Total	2·41	1071	551		
	Ratio to dairy	5·86	2·45	2·20		
Scenario 3. Optimised for smallest amount of foods by weight						
A. WWEIA consumption constraint: none						
4602	Ready-to-eat cereal, higher sugar (>21·2 g/100 g)	0·49	1205	77	1·3–1·9	2·2–3·3
5404	Nutrition bars	0·93	979	64	1·6	2·7
	Total	1·42	2184	141		
	Ratio to dairy	3·46	4·99	0·56		
B. WWEIA consumption constraint: 90th percentile of non-dairy foods						
4602	Ready-to-eat cereal, higher sugar (>21·2 g/100 g)	0·49	1205	77	1·3–1·9	2·2–3·3
5404	Nutrition bars	0·93	979	64	1·6	2·7
	Total	1·42	2184	141		
	Ratio to dairy	3·46	4·99	0·56		

WWEIA, What We Eat In America.

* In addition to excluding dairy products from the WWEIA database (see Table 1), foods and beverages that are not reasonable dairy alternatives on a population basis and beverages with virtually no energy content, but that contain small amounts of micronutrients, also were excluded: WWEIA Food Category (category codes): Baby foods (9002, 9004, 9006, 9008, 9010, 9012, 9202), infant formulas (9402, 9404), protein and nutritional powders (9802), nutritional beverages (7208)_,_ coffee (7302), tap water (7702), bottled water (7704), diet soft drinks (7102) and other diet drinks (7106).

## Results

The optimised food combinations varied considerably in the two phases, the three optimisation priorities and under the two consumption constraints (Tables 4 and 5). All met or exceeded the target levels of protein and shortfall nutrient in a USDA cup-equivalent of dairy (see online supplementary material, Supplemental Tables 1 and 2).

### Phase 1

In phase 1, only dairy foods, including the dairy component in mixed dishes, were excluded from the WWEIA data source of potential non-dairy foods (Table 1). The results for each of the three optimisation priorities are shown in Table 4.

#### Scenario 1. Optimised for lowest cost

Whether or not constrained at the 90th percentile of WWEIA food intake, the cost and energy content of the optimised food combinations were higher than for a USDA serving of dairy. The gram amount was 11–20 times larger than a USDA serving of dairy. This was due to unreasonably large amounts of tap water (about 7–14 twelve-oz servings) selected by the model as a low-cost source of two nutrients, Ca and Mg, accounting for one-fourth to one-half of that in a USDA serving of dairy (see online supplementary material, Supplemental Table 1). These findings led to excluding water as a dairy substitute in phase 2.

#### Scenario 2. Optimised for fewest kJ

Whether or not constrained at the 90th percentile of WWEIA food intake, the optimised food combinations would provide a similar amount of energy to that in one USDA cup-equivalent serving of dairy (410 *v*. 435 kJ, respectively), but at a higher cost (2·4–4·0 times greater) and with 22–41 times more food by weight. This also is due to large amounts of water (bottled and/or tap water; about 7–27 twelve-oz servings) that account for 92–96 % of Ca and 77–84 % of Mg (see online supplementary material, Supplemental Table 1). These findings further contributed to excluding water, both tap and bottled water, as potential non-dairy foods in phase 2.

#### Scenario 3. Optimised for smallest amount of food by weight

Whether or not constrained at the 90th percentile of WWEIA intake, the total gram amount of the food combinations was 44 % less than that of a USDA cup-equivalent of dairy, but the energy content was five times greater and costs 3·5 times higher. Although the amount of food by weight was less than one USDA serving of dairy, on a RACC serving basis, one would need to consume about 1·3–1·9 daily servings of *higher-sugar ready-to-eat cereal* and 1·6 *nutrition bars* to replace the protein and shortfall nutrients in one USDA dairy serving.

### Phase 2

The optimisation parameters in phase 2 were the same as in phase 1, except that foods and beverages that are not reasonable dairy alternatives on a population basis and beverages with virtually no energy, but that contain small amounts of micronutrients, were excluded as food options in the linear programming modelling (Table 2). The results for the three scenarios are shown in Table 5.

#### Scenario 1. Optimised for lowest cost

The optimised food combinations would cost around 40 % more than that of one USDA cup-equivalent of dairy. Whether or not consumption of the WWEIA food categories was constrained at the 90th percentile of intake, r*olls and buns* and *higher sugar ready-to-eat cereal* together account for most of the cost. The gram amount of the food combinations was the same as or 15 % less than that of one USDA serving of dairy, but on a RACC serving basis, a large amount of food would need to be consumed at six times more energy.

In diets without dairy foods, to replace the shortfall nutrients in the current 1·8 dairy servings, one would need to consume 3·6 *rolls and buns* and 2·0–3·0 servings of *higher sugar ready-to-eat cereal* plus four fluid ounces of *citrus juice* and a small amount of *eggs and omelettes.* These foods together would cost only 34 cents more per day but would contribute 4502 kJ to the daily intake; that is, 3720 more kJ than 1·8 USDA servings of dairy. At the recommended three USDA servings of dairy for ages ≥9 years, even larger amounts of food and energy (approximately 7531 kJ/d) would be needed.

#### Scenario 2. Optimised for fewest kJ

The optimised food combinations would provide more than twice the energy, about twice the amount of food by weight, and cost 6−8 times more than a USDA cup-equivalent of dairy. *Fish*, *dark green vegetables* (*excluding lettuce*) and *citrus juice*, when included, account for 93 % of the energy (kJ). Only two foods, *fish* and *dark green vegetables* (*excluding lettuce*), were included in the optimised food combination when consumption of the WWEIA food categories was not constrained, and when constrained at the 90th percentile of WWEIA consumption, *lettuce and lettuce salads* and *citrus juice* also were included.

In diets without dairy foods, to replace the shortfall nutrients in the current 1·8 dairy servings/d, one would need to consume 0·8 servings of *fish,* 4·0 servings of *dark green vegetables* (*no lettuce*), 2·2 servings of *lettuce/lettuce salads* and 11·2 fluid ounces of *citrus juice.* These foods would contribute 1146 more kJ and cost $US 3·60 more per day than the corresponding 1·8 USDA servings of dairy. At the equivalent of the recommended three USDA servings of dairy for ages ≥9 years, one would need to consume 40 % more of these foods at 3213 daily kJ and a daily cost of $US 7·23.

#### Scenario 3. Optimised for smallest amount of foods by weight

When optimised for the smallest amount of food by weight, the results were the same as in phase 1, scenario 3 (Tables 4 and 5). The gram amount would be 44 % less than in a single USDA cup-equivalent of dairy, but substantial amounts of *higher sugar ready-to-eat cereal* and *nutrition bars* would need to be consumed at five times more energy and 3·5 times greater cost.

In diets without dairy, to replace the shortfall nutrients in the current 1·8 dairy servings per day, one would need to eat 2·2–3·3 servings of *higher sugar ready-to-eat cereal* and 2·7 *nutrition bars* at 3933 kJ and a cost of $US 2·56/d. At the recommended three dairy servings per day for ages ≥9 years, consumption amounts would be 40 % greater at around 4–6 servings of *higher sugar ready-to-eat cereal* and five *nutrition bars* at about 6276 kJ and a cost of $US 4·26/d.

### Milk substitutes

Many of the food combinations included *ready-to-eat cereals (higher sugar),* which typically are consumed with milk or yogurt. Although the *milk substitutes* WWEIA category would be a logical replacement for dairy, especially milk, this category was not among the foods selected by the linear programming optimisation models. To better understand why the linear programming models did not include *milk substitutes* in any non-dairy food combinations, we compared the energy (kJ), key nutrient levels and cost of the *milk substitutes* WWEIA category with that of a USDA cup-equivalent of dairy. When we matched the Ca levels of the *milk substitutes* with the amount in a USDA cup-equivalent of dairy, several nutrient levels were lower (vitamin D, 22 % lower; vitamin A, 23 % lower; Mg, 16 % lower; choline, 42 % lower; K, 50 % lower; protein, 66 % lower), and although the energy content was 22 % lower, the cost was 30 % higher.

### Limiting nutrients

The limiting nutrients in all of the non-dairy food combinations were vitamin D and Ca whether optimised for the lowest cost, fewest number of kJ or the smallest amount of food by weight. The non-dairy food sources of Ca and vitamin D came from a limited number of foods and varied depending on the optimisation priority.

### Nutrients to limit

The 2015–2020 DGA classifies added sugars (<10 % of energy intake/d), Na (<2300 mg/d), saturated fat (<10 % of energy intake/d) and *trans*-fats as *nutrients to limit* to help individuals achieve healthy eating patterns^(2)^. In some of the optimised food combinations, Na, added sugars and/or saturated fat were higher than in a USDA cup-equivalent of dairy (see online supplementary material, Supplemental Tables 1 and 2). In phase 2, the optimised food combinations with *higher sugar ready-to-eat cereals* contained 5·2–5·7 tsp of added sugars (*v*. none in USDA cup-equivalents of dairy). The food combinations with *rolls and buns, higher sugar ready-to-eat cereal, fish, dark green vegetables* (*no lettuce*) and*/*or *nutrition bars* were major sources of Na, which at 532–903 mg is 4·0–6·8 times higher than in a USDA serving of dairy. Saturated fat in all the optimised food combinations was 1·3–2·0 times higher than in USDA servings of dairy.

## Discussion

We show that combinations of non-dairy foods identified using linear programming optimisation methodology could replace the protein and shortfall nutrients in dairy, but not without negative impacts on cost, energy intake and the need to consume large amounts of food. When we limited food amounts to the 90th percentile of average food intakes, all food combinations identified through optimisation require large amounts of food, contain more than twice the energy and cost up to nearly six times more than USDA servings of dairy – regardless of whether optimised for lowest cost, the fewest number of kJ or the smallest amount of food by weight (Table 5).

Only twelve out of the approximately 150 possible WWEIA food categories were represented in the optimised food combinations (Tables 4 and 5). After the removal of the food categories that were unreasonable sources of nutrients on a population basis (Table 2), only eight WWEIA categories were represented (Table 5). Among these, some (e.g. *fish; dark green vegetables*) are naturally rich sources of one or more of the shortfall nutrients in dairy, and others (e.g., *ready-to-eat cereal, high sugar; citrus juice; nutrition bars*) are fortified with one or more of these nutrients (see online supplementary material, Supplemental Table 2).

*Meat* and other animal-based foods, which are rich sources of protein, were not represented in the optimised food combinations; only *fish* and in some scenarios also small amounts of *eggs and omelettes* were included as sources of protein and shortfall nutrients. Another WWEIA category, *milk substitutes,* which includes soya, almond, rice and coconut beverages, also was not among the foods in the optimised food combinations. Although they are infrequently consumed in the USA (<0·1 cup-equivalent per day)^(32)^, they are designed to mimic the nutrient content of dairy milk and were among the 150 possible WWEIA food categories available for selection in all linear programming optimisation scenarios. As noted above, differences in their nutrient profiles from that in the dairy^(33,34)^ and their cost may explain their absence in the optimised food combinations. When we matched the Ca levels of *milk substitutes* with that in a USDA cup-equivalent of dairy, the levels of protein and several of the shortfall nutrients were 22–66% lower in *milk substitutes*, and although the energy content was 22 % lower, the cost was 30 % higher than that of a USDA cup-equivalent of dairy.

In all optimised food combinations, both vitamin D and Ca levels were equal to those in a USDA cup-equivalent of dairy and therefore were the limiting nutrients for these analyses. Dairy and some foods in the optimised food combinations (i.e. *fish*, *ready-to-eat cereals*, *orange juice*) are among the top food sources of Ca and/or vitamin D in the USA^(2,14)^. In the USA, vitamin D fortification of milk is mandatory by law and voluntary for yogurt and cheese^(35)^. Vitamin D fortification is also permitted for only a select few non-dairy foods including Ca-fortified fruit juices, breakfast cereals and ‘beverages made from edible plants intended as milk alternatives, such as beverages made from soya, almond and coconut’. In the optimised food combinations, *fish,* c*itrus juice* and/or *higher sugar ready-to-eat cereals* accounted for essentially all (>99 %) of the vitamin D, and *citrus juice, higher sugar ready-to-eat cereals, dark green vegetables (no lettuce), rolls and buns* and *nutrition bars* were the major non-dairy sources of Ca (see online supplementary material, Supplemental Tables 1 and 2).

Vitamin D and Ca have been identified as limiting nutrients in other studies examining dietary patterns that are nutritionally adequate, affordable, culturally acceptable and/or have lower environmental impacts than current eating patterns (see reviews (15,16)). The criteria for nutritional adequacy, however, have been inconsistent across studies^(15)^. A full complement of macro- and micro-nutrients (29–39 nutrients) was used to establish nutritional adequacy in studies from Denmark^(22)^, the Netherlands^(19,21,24)^, France^(23,36^, and the USA^(26)^, although with varying levels for some nutrients. For example, the minimum value for vitamin D ranged from 2·5 to 15 µg/d and for Ca from 800 to 1000 mg/d^(15,19,21–24,26,36)^. Other limiting nutrients included vitamin A, K, vitamin C and/or fibre. Relaxation of constraints such as the dietary cost, deviation from usual food habits and/or percentage reduction in environmental impact was required to achieve nutritional adequacy in some studies^(21–24)^. In others, nutrient adequacy was established with fewer nutrients (14–22 nutrients), lower values for some nutrients and/or the absence of limiting nutrients such as vitamin D, fibre and/or K^(17,18,20,25)^. These studies indicated that nutritionally adequate, affordable diets with lower environmental impacts could be achieved with minimal shifts in commonly consumed foods. Differences in nutrient recommendations across the globe and the variability in criteria for nutritional adequacy among studies likely impede broad conclusions for dietary recommendations from the totality of the current research.

Cost and taste rank at the top of many factors involved in making food choices^(3,37)^. The higher cost of fruits, vegetables, lean meats and fish, unfortunately, may be a barrier to eating healthier diets for many, especially low-income groups^(3,28)^. The cost of the three Healthful Diet Patterns exemplified in the 2015–2020 DGA, for example, is at least 50 % more than that of the average US diet^(28)^. The higher cost of the optimised food combinations in our study (41 % to eight times more than dairy) appears to be driven by the large amounts of foods needed to replace the vitamin D and Ca in a USDA serving of dairy. This may be explained in part because milk, a low-cost source of these two nutrients^(38)^, accounts for 53 % of USDA cup-equivalent servings of dairy.

Dairy food consumption is recommended by most food-based dietary guidelines around the world^(39)^. Dairy not only is an important dietary source of key nutrients, including nutrients of public health concern, but also has been associated with beneficial or neutral effects on risk of certain chronic diseases, including CVD, hypertension and type 2 diabetes^(14,40–43)^. Following national and international guidelines for daily servings of dairy, which in some guidelines is preferentially for lower fat dairy foods, is important for all population groups. Nonetheless, there continues to be much discussion and debate about climate and other environmental impacts from animal agriculture while ensuring food security for a projected global population of 9–10 billion by 2050^(8,10,15,16,44)^. Removing animal products, including dairy, from the diet while maintaining nutrient adequacy, affordability and minimising environmental impacts has been advocated by some as achievable and necessary^(8–10)^ despite conflicting and limited evidence^(24,26)^. Although removing animal foods from the diet may lead to lower greenhouse gas emissions, trade-offs such as nutritional inadequacies, substantial deviations from familiar eating patterns, increased energy intake and/or higher diet costs are counter to public health initiatives to help consumers develop healthy, culturally acceptable and affordable dietary patterns that meet nutrient recommendations without exceeding energy needs^(2)^.

## Limitations

Our study had several limitations. First, we did not account for the bioavailability of the plant-based or fortified food sources in the optimised food combinations; thus, the contribution of Ca in *dark green vegetables* (*no lettuce*) or Ca and vitamin D in fortified *citrus juice* and *high-sugar ready-to-eat cereals* may have been overestimated. Nonetheless, our study provides insights into the types of foods and likely reasonable estimates of the quantities of WWEIA foods needed to replace shortfall nutrients in USDA servings of dairy. Second, we did not include nutrient constraints for the nutrients classified as ‘nutrients to limit’ by the 2015–2020 DGA, specifically saturated fat, Na and added sugars. We surmise that the high Na levels in the optimised food combinations are explained by the large amounts of food needed. Future optimisation studies with constraints also for ‘nutrients to limit’ would provide valuable insights. Third, although the *milk substitutes* WWEIA category would be a logical replacement for dairy, especially milk, *milk substitutes* were not forced *a priori* in the optimised food combinations in any of the linear programming models. This could also be examined in future optimisation studies. Fourth, the NHANES data source for the WWEIA food categories was based on dietary 24-h recall, and therefore, the consumption amounts may not be fully representative of usual eating habits. Last, as has been described elsewhere^(28)^, attaching retail prices to WWEIA food consumption provides an estimate of the cost of each food category, but this may not be a complete representation of actual food expenditures due to assumptions inherent in the development of the USDA food price database^(28)^. Even with these limitations, the current study provides insights into the relative costs and food combinations that can serve as sources of the protein and shortfall nutrients when the dairy group is excluded from eating patterns.

## Conclusion

Healthy eating patterns are composed of a variety of foods that provide the nutrients needed for health, help reduce chronic disease risk and support a healthy body weight^(2)^. Identifying foods to replace key nutrients when excluding or severely limiting the consumption of specific food groups, such as dairy, is critical. The non-dairy food combinations identified using linear programming optimisation are not reasonable substitutes for the protein and shortfall nutrients in diets without dairy. Whether replacing the 1·8 servings of dairy in current diets or at the DGA recommended three daily servings for ages ≥9 years, the large amounts of food, greater amount of energy (41 % to eight times more kJ) and a higher cost (2·3–6·3 times more) seem untenable. Identifying affordable, consumer-acceptable foods that can be reasonably integrated into the diet to replace key nutrients provided by dairy is critical for dietary patterns without this important food group.

## Supplementary material

For supplementary material accompanying this paper visit https://doi.org/10.1017/S1368980020001937 click here to view supplementary material
